# Trend of oral and pharyngeal cancer mortality in Brazil in the period of 2002 to 2013

**DOI:** 10.11606/S1518-8787.2018052000251

**Published:** 2018-01-29

**Authors:** Lillia Magali Estrada Perea, Marco Aurélio Peres, Antonio Fernando Boing, José Leopoldo Ferreira Antunes

**Affiliations:** I Universidade Federal de Santa Catarina. Programa de Pós-Graduação em Saúde Coletiva. Florianópolis, SC, Brasil; II University of Adelaide. Adelaide Dental School. Australian Research Center for Population Oral Health. Adelaide, Australia; III Universidade Federal de Santa Catarina. Departamento de Saúde Pública. Florianópolis, SC, Brasil; IV Universidade de São Paulo. Faculdade de Saúde Pública. Departamento de Epidemiologia. São Paulo, SP, Brasil

**Keywords:** Mouth Neoplasms, mortality, Pharyngeal Neoplasms, mortality, Mortality, trends, Brazil, Neoplasias Bucais, mortalidade, Neoplasias Faríngeas, mortalidade, Mortalidade, tendências, Brasil

## Abstract

**OBJECTIVE:**

To analyze the trend of oral and pharyngeal cancer mortality rates in the period of 2002 to 2013 in Brazil according to sex, anatomical site, and macroregion of the country.

**METHODS:**

The mortality data were obtained from the Mortality Information System and the population data were obtained from the Brazilian Institute of Geography and Statistics. The trend of the rates standardized by sex and age was calculated using the Prais-Winsten estimation, and we obtained the annual percentage change and the respective 95% confidence intervals, analyzed according to sex, macroregion, and anatomical site.

**RESULTS:**

The average coefficient of oral cancer mortality was 1.87 per 100,000 inhabitants and it remained stable during the study period. The coefficient of pharyngeal cancer mortality was 2.04 per 100,000 inhabitants and it presented an annual percentage change of -2.6%. Approximately eight in every 10 deaths occurred among men. There was an increase in the rates of oral cancer in the Northeast region (annual percentage change of 6.9%) and a decrease in the Southeast region (annual percentage change of -2.9%). Pharyngeal cancer mortality decreased in the Southeast and South regions with annual percentage change of -4.8% and -5.1% respectively. Cancer mortality for tonsil, other major salivary glands, hypopharynx, and other and unspecified parts of mouth and pharynx showed a decreasing trend while the other sites presented stability.

**CONCLUSIONS:**

Pharyngeal cancer mortality decreased in the period of 2002 to 2013. Oral cancer increased only in the Northeast region. Mortality for tonsil cancer, other major salivary glands, hypopharynx, and other and ill-defined sites in the lip, oral cavity, and pharynx decreased.

## RESUMO

**OBJETIVO:**

Analisar a tendência das taxas de mortalidade por câncer de boca e de faringe no período de 2002–2013 no Brasil segundo sexo, sítio anatômico e macrorregião do país.

**MÉTODOS:**

Os dados sobre mortalidade foram obtidos junto ao Sistema de Informações sobre Mortalidade e os dados das populações foram obtidos junto ao Instituto Brasileiro de Geografia e Estatística. A tendência das taxas padronizadas por sexo e faixa etária foi calculada por regressão de Prais-Winstein, obtendo a sua variação percentual anual e os respectivos intervalos de confiança de 95%, analisados segundo sexo, macrorregião e sítio anatômico.

**RESULTADOS:**

O coeficiente médio de mortalidade por câncer de boca foi de 1,87/100.000 habitantes e permaneceu estável durante o período estudado. O coeficiente de mortalidade por câncer de faringe foi de 2,04/100.000 habitantes e apresentou variação percentual anual de -2,6%. Aproximadamente oito em cada 10 óbitos ocorreram entre homens. Observou-se aumento nas taxas por câncer de boca na região Nordeste (variação percentual anual de 6,9%) e diminuição na região Sudeste (variação percentual anual -2,9%). Mortalidade por câncer de faringe diminuiu nas regiões Sudeste e Sul com variação percentual anual de -4,8% e -5,1% respectivamente. Mortalidade por câncer de amígdala, outras glândulas salivares maiores, hipofaringe e outras partes não especificas de boca e faringe, apresentaram tendência de declínio enquanto os demais sítios apresentaram estabilidade.

**CONCLUSÕES:**

A mortalidade por câncer de faringe apresentou diminuição no período 2002–2013. O câncer de boca apresentou aumento só na região Nordeste. Mortalidade por câncer de amígdala, outras glândulas maiores, hipofaringe e outras localizações mal definidas de lábio, cavidade oral e faringe mostraram declínio.

## INTRODUCTION

Oral and pharyngeal cancer is considered a global public health problem^1^. According to the International Agency for Research on Cancer^a^ (IARC), 571,386 new cases of the disease were diagnosed worldwide and 316,168 deaths were recorded in 2015. The literature estimates that these figures will increase in coming years, reaching 350,000 deaths in 2020, and resulting in a rate of 4.7 deaths per 100,000 inhabitants.

The mortality trend from this disease varies by region. In Europe, oral and pharyngeal cancer mortality rates have been decreasing since the 1970s^2^, while in Oceania^3^ and in several Latin American countries, these rates have been increasing since the 1980s^4^. Brazil is the country with the highest mortality rates for both sites in Latin America^5^, showing an increase since the 1980s^6^.

There are several factors that may influence the trend of mortality rates, such as changes in the prevalence of exposure to the main risk factors of the disease^7^ ^,^ ^8^ and the availability and access to diagnosis and early treatment. Significant variations have occurred in these factors in the Brazilian context since the last decades of the twentieth century.

According to data from the Observatory of the Brazilian Tobacco Control Policy (INCA)^b^, there was a 46% decrease in the percentage of smokers between 1989 and 2013. On the other hand, alcohol consumption, which was increasing since 1960, has stagnated since 2000^9^. Human papillomavirus (HPV) has also been associated with the carcinogenesis of this disease, especially with oropharyngeal and tonsil cancer^10^. However, these changes have the potential to modify or reduce rates only in the long term, since the effects of carcinogenic factors are cumulative and of long latency.

Another important factor that may influence the trend of mortality is the early diagnosis of the disease and the provision of adequate therapy. Diagnosis in the late stages implies a worse prognosis and a decrease in the survival rate^11^. Similarly, the advances in the techniques used in the treatment can also influence the quality of life of the patient and avoid possible relapses that end in death^12^.

Boing et al.^6^ have analyzed the trends of oral and pharyngeal cancer mortality rates between 1979 and 2002 in Brazil and they have observed greater reduction in values for anatomical sites that are more accessible to clinical inspection, which suggests a possible connection between the visual facility for diagnostic examination and lower mortality rates. However, according to an electronic search conducted in the bibliographic databases SciELO, PubMed, Lilacs, and Scopus, we found no other trend studies since that last study that allow us to analyze the evolution of oral and pharyngeal cancer mortality rates according to specific anatomical site.

Brazil is in a process of demographic and epidemiological transition that has been directly affecting the incidence and mortality of chronic non-communicable diseases^1^. This scenario demands studies, follow-up, and analysis of these diseases, among them, oral and pharyngeal cancer.

The objective of this study was to analyze the trends of oral and pharyngeal cancer mortality in Brazil according to anatomical site, sex, and macroregion of the country during the period of 2002 to 2013.

## METHODS

In order to analyze the temporal trend of oral and pharyngeal cancer mortality in Brazil, we carried out an ecological study using data from the deaths in the country during the period of 2002 to 2013. The annual mortality data were obtained from the Mortality Information System (SIM)^c^, available in the website of the Information System of the Brazilian Unified Health System (DATASUS). The number of inhabitants is provided by the Brazilian Institute of Geography and Statistics (IBGE)^d^ and originated from the 2010 Census and from intercensorial estimates for the remaining years. The deaths with ignored sex and age were excluded from this analysis and amounted to 0.05% of the cases.

We analyzed the deaths from oral cancer (C00.0–C08.9) and pharyngeal cancer (C09–C14.8) according to the International Classification of Diseases, Tenth Revision (ICD-10), and according to macroregion of residence (North, Northeast, Southeast, South, and Midwest), sex, and anatomical site. We also analyzed the temporal trend of mortality rates for each anatomical site, which were grouped according to common characteristics related to tissue location and histology. This procedure ensured a sufficient number of cases and provided greater stability for the analyses.

We calculated the oral and pharyngeal cancer mortality rates per 100,000 inhabitants and adjusted them using the direct method by sex and age groups (with intervals of five years). We considered as the standard the percentage distribution of the world population provided by the World Health Organization^13^ (2001), applied to the total world population of 2015. We carried out this procedure to guarantee the comparison of the results with previous studies that use other populations for standardization.

To calculate the annual percentage variation (APC) of the rates, we used the Prais-Winsten regression, which predicts first-order autocorrelation correction. The dependent variable was the logarithm of the rates, and the independent variable was the years of the historical series. The calculation of the annual percentage change of the rates was performed with the following formulas, as suggested by Antunes and Waldman^14^.

(1) $$-1+10^{b}=\Delta$$

For the calculation of the confidence intervals:

(2) $$\Delta_{95\;Cl}=-1+10^{\left(b\pm t*se\right)}$$

Where “*b*” corresponds to the annual growth rate. The values of “*b*” and standard deviation (*se*) were extracted from the regression analysis, and the value of “*t*” is supplied by the Student's distribution table *t*. The trend of increase, decrease, or stagnation was expressed as APC, with the respective confidence intervals (95%), and we considered as stationary the trend whose regression coefficient was not different from zero (p > 0.05).

In order to facilitate the visualization of the trends, we reduced the white noise in the graphs of the historical series, using the technique of 3rd order centered moving averages. The trend analysis was performed in the Stata program, version 13.

## RESULTS

Between 2002 and 2013, there were 74,342 deaths from oral cancer (n = 35,534) and pharyngeal cancer (n = 38,808) in Brazil, corresponding to 3.9% of the deaths from all neoplasms in the period studied. Approximately eight in every 10 deaths occurred among men.

When analyzed together, the oral and pharyngeal cancer mortality rates presented stability in both men, with average coefficient of 6.74 per 100,000 inhabitants, and women, with average coefficient of 1.45 per 100,000 inhabitants. Although with a stable trend, men had an average coefficient that was 4.6 times higher than women. The ratio between the average male and female coefficient was 3.7:1 for oral cancer and 6:1 for pharyngeal cancer. This ratio decreased in 1.5% and 1.6% for oral and pharyngeal cancer mortality, respectively, when we compared the ratio of male and female coefficient of 2002 to that of 2013 (data not shown).

**Figure 1:**
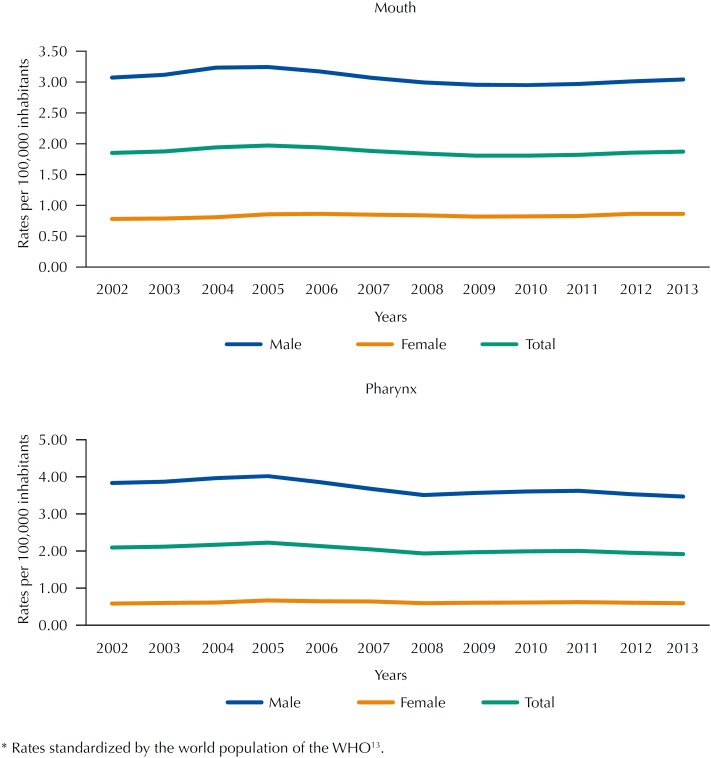
Historical series of oral and pharyngeal cancer mortality rates* according to sex. Brazil, 2002–2013.

When analyzing the oral and pharyngeal cancer mortality rates separately, we observed stability in oral cancer mortality (average coefficient of 1.87 per 100,000 inhabitants) and decrease in pharyngeal cancer mortality (average coefficient of 2.4 per 100,000 inhabitants), especially among men (APC of -2.7%). Figure 1 shows the behavior of the oral cancer and pharyngeal cancer mortality rates according to sex.

Oral cancer and pharyngeal cancer presented marked differences according to regions (Table 1). For oral cancer, the Northeast region presented one of the lowest average coefficients for the period (1.60 per 100,000 inhabitants), but it had an annual average increase in mortality of 6.9%. On the other hand the Southeast region presented the second highest average coefficient (2.04 per 100,000 inhabitants); however, it was the only region with a reduction in mortality rates (APC of -2.9%).

**Table 1:** Number and percentage of deaths, mean coefficient per 100,000 inhabitants, and trend of oral and pharyngeal cancer mortality rates according to regions. Brazil, 2002–2013.

Region	Deaths		Average Coefficient^a^	APC^b^	95%CI^c^	Interpretation
	n	%				
Mouth						
North	1,170	3.3	1.2	5.0	-0.9–11.3	Stable
Northeast	8,012	22.5	1.6	6.9	1.5–12.6	Increase
Southeast	18,007	50.7	2.0	-3.0	-4.6– -1.4	Decrease
South	6,331	11.9	2.1	-2.8	-6.4–0.9	Stable
Midwest	2,014	5.7	1.8	-3.6	-8.7–1.8	Stable
Total	35,534		1.9	-0.6	-2.6–1.5	Stable
Pharynx						
North	1,185	3.1	1.1	1.9	-1.8–5.8	Stable
Northeast	7,549	19.4	1.5	5.9	-0.4–12.5	Stable
Southeast	20,021	51.6	2.3	-4.9	-7.9– -1.8	Decrease
South	7,458	19.2	2.5	-5.1	-7.9– -2.3	Decrease
Midwest	2,595	6.7	2.2	-4.9	-9.7–0.3	Stable
Total	38,808		2.0	-2.7	-5.0– -0.3	Decrease

a Rates standardized by the world population, WHO^13^.

b Annual percentage change.

c Confidence interval of the APC.

**Figure 2:**
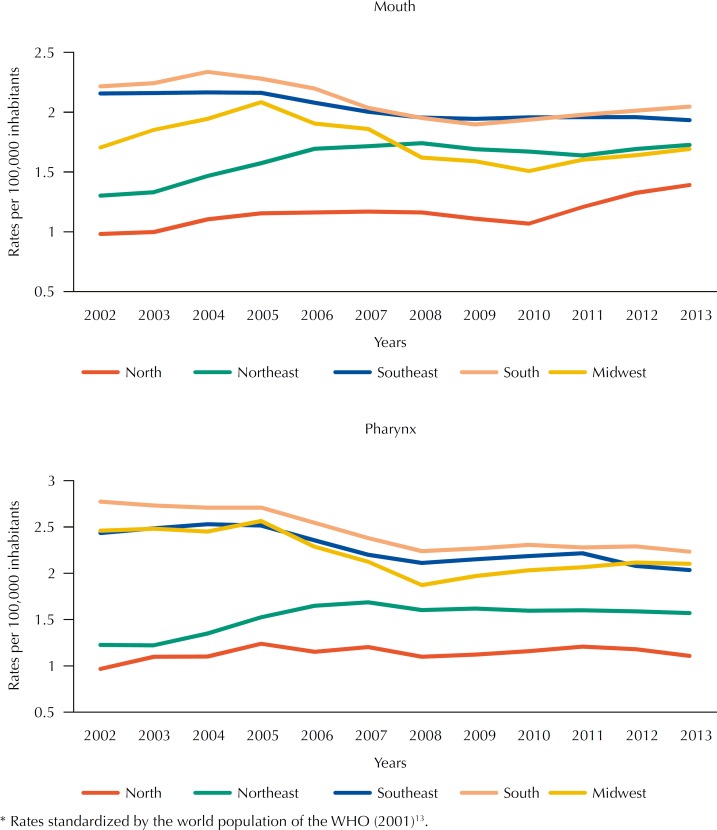
Historical series of oral and pharyngeal cancer mortality rates* according to regions, Brazil, 2002–2013.

As for pharyngeal cancer, both the Southeast region and the South region presented a decrease in mortality rates, with APC of -4.8% and -5.1%, respectively (Table 1). The values were stable in the other regions. Figure 2 presents the historical series of oral and pharyngeal cancer mortality rates according to regions.

The anatomical site with the highest average coefficient in the period studied was the oropharynx (0.91 per 100,000 inhabitants), followed by other and unspecified parts of mouth (0.62 per 100,000 inhabitants). We observed decreasing trends for cancer of tonsil (-6.6%), other major salivary glands (-4.9%), hypopharynx (-5.8%), and other and unspecified sites of mouth and pharynx (-3.8%). The trends of mortality rates in the other sites were stable during the study period. Table 2 describes the number of deaths, the average coefficient, and the APC of the oral and pharyngeal cancer mortality rates according to anatomical site.

**Table 2:** Number and percentage of deaths, mean coefficient per 100,000 inhabitants, and trend of oral and pharyngeal cancer mortality rates according to anatomical site. Brazil, 2002–2013.

ICD-10	Deaths		Average Coefficient^a^	APC^b^	95%CI^c^	Interpretation
	n	%				
Anatomical site						
C00 – Lip	589	0.8	< 0.1	-0.1	-7.5–7.8	Stable
C01 – Base of tongue	4,491	6.0	0.2	2.4	-0.9–5.9	Stable
C02 – Other and unspecified parts of tongue	10,326	13.9	0.5	-0.4	-2.5–1.8	Stable
C03 – Gum	473	0.6	< 0.1	-0.5	-5.1–4.3	Stable
C04 – Floor of mouth	2,040	2.7	0.1	2.7	-0.3–5.8	Stable
C05 – Palate	2,650	3.6	0.1	0.7	-2.2–3.6	Stable
C06 – Other and unspecified parts of mouth	11,683	15.7	0.6	-2.5	-5.3–0.5	Stable
C07 – Parotid gland	2,489	3.4	0.1	-1.6	-6.3–3.3	Stable
C08 – Other and unspecified major salivary glands	793	1.1	< 0.1	-5.0	-9.5– -0.	Decrease
C09 – Tonsil	1,705	2.3	0.1	-6.7	-9.4– -3.	Decrease
C10 – Oropharynx	17,292	0.2	0.9	-0.7	-3.4–2.0	Stable
C11 – Nasopharynx	3,181	4.3	0.2	-0.7	-2.9–1.6	Stable
C12 – Pyriform Sinus	787	1.1	< 0.1	-3.2	-8.4–2.4	Stable
C13 – Hypopharynx	4,789	6.4	0.3	-5.9	-9.5– -2.	Decrease
C14 – Other and ill-defined sites in the lip oral cavity, and pharynx	11,054	14.9	0.6	-3.8	-6.4– -1.	Decrease
Grouped sites						
C01 + C02 – Tongue	14,817		0.8	0.4	-1.2–2.0	Stable
C07 + C08 – Parotid gland and other and unspecified major salivary glands	3,282		0.2	-2.8	-6.0–0.4	Stable
C09 + C10 – Tonsil and oropharynx	18,997		1.0	-1.3	-3.6–1.1	Stable

a Rates standardized by the world population, WHO^13^.

b Annual percentage change.

c Confidence interval of the APC.

## DISCUSSION

The temporal trends of oral and pharyngeal cancer mortality rates in Brazil in the period of 2002 to 2013 showed different patterns according to sex, regions of the country, and anatomical sites of the disease. According to sex, although stable, the oral and pharyngeal cancer mortality rates were higher in males than in females throughout the study period. However, the ratio between the sexes presented a decrease throughout the historical series investigated, which may be related to the acquisition of habits associated to the masculine lifestyle by the women.

This discrepancy in the rates between sexes is observed both internationally^14^ and in Brazil^15^ and it is possibly related to the exposure in the past to the main risk and protection factors, such as tobacco, whose prevalence of consumption in Brazil is higher in men than in women^16^. Another possible explanation for this discrepancy between the sexes is the frequency of regular dental appointment, which is usually higher in women than in men^17^, and the socioeconomic differences between genders^18^.

In the analysis by anatomical site, we observed a decrease in cancer mortality for tonsil, other major salivary glands, hypopharynx, and unspecified parts of mouth and pharynx in Brazil. We observed stability of the trends of the rates of the other anatomical sites, being some of them difficult to diagnose or of high incidence. Oropharynx, for example, previously considered as an anatomical site with a growing trend^19^, presented stability in the period of 2002 to 2013. Nevertheless, it was the anatomical site with the highest average coefficient per 100,000 inhabitants in the studied period.

The stagnation or stability observed in these later parts, which are considered difficult to inspect on clinical examination, may be a consequence of improved access to health services and more conservative and effective surgical techniques^20^.

In this study, we observed differences between macroregions. The observed increase in the trend of oral cancer mortality in the Northeast region suggests that there was an improvement in the information systems in the study period, which could lead to a higher number of death records for the calculation^21^. However, this increase may also be related to changes in lifestyles, increasing exposure to risk factors. The improvement of the information system may have partially influenced the observed increase in the rates; therefore, this result must be interpreted with caution. The other regions of the country presented stability in the rates investigated.

The difference in the trends of the rates between regions may also have been influenced by access to health services, since the North and Northeast regions have the lowest rates of active health professionals and the highest percentage of persons who report having never consulted a dentist^22^. Regular appointments with the health professional, which influences the timely diagnosis of precancerous lesions, also presents great variations among regions, which may explain the different behavior of the oral and pharyngeal cancer mortality rates in each one of them, as richer regions have a higher prevalence of dental appointments^23^.

As in the study on the trends of oral and pharyngeal cancer mortality in Brazil for the period of 1979 to 2002, the trend of oral cancer mortality in Brazil between 2002 and 2013 presented stability for both sexes. However, Boing et al.^6^ have observed an increase in pharyngeal cancer mortality, whereas this rate decreased in the period of 2002 to 2013. Similarly, cancer mortality for oropharynx, hypopharynx, and other undefined parts showed an increase in the period of 1979 to 2002, while we observed stability for these specific anatomical sites in the 12 years considered in our study.

The oral and pharyngeal cancer mortality rates were higher in the South and Southeast regions of the country, both in the results observed by Boing et al.^6^ and in this study. We know that tobacco is the main risk factor for oral and pharyngeal cancer. The South and Southeast regions present a higher prevalence of tobacco consumption than the other regions^24^, which may be affecting the mortality rates in these regions.

The study of trends in cancer mortality is complex and its analysis should consider that the potentially beneficial consequences of health interventions will only have an effect in the long term given the cumulative action of risk factors. Between 1989 and 2010, the drop in the percentage of smokers in Brazil was 46%^16^. Therefore, it is expected that its effects are being reflected in the reduction of mortality observed today, especially in the adult persons who, for years, have been affected by the effects of decreasing tobacco consumption.

As a possible consequence of public policies aimed at tobacco and alcohol control, the country has experienced changes in the consumption of these substances, which may reflect in the different patterns of the trends of oral and pharyngeal cancer mortality. The decrease observed in the country in smoking prevalence^16^ may influence the long-term trend of oral and pharyngeal cancer mortality rates in different anatomical sites.

It is important to note that, while the incidence of cancer can be controlled from primary prevention, mortality is susceptible to secondary prevention from timely diagnosis and tertiary prevention, which seeks to limit damage, control pain, prevent secondary complications, and improve the quality of life during treatment^25^. As there is no scientific evidence that a visual examination as part of a population-based screening program reduces the oral and pharyngeal cancer mortality rate, authors have suggested that strategies focused on individuals exposed to major risk factors could result in a more effective secondary prevention^26^.

As any study with secondary information, the results shown here depend on the accuracy and completeness of information systems. The quality and coverage of the mortality data provided by the SIM have gradually increased since its decentralization in 1992, as well as the adequate filling of the data, which is approximately 90%^27^. In Brazil, coverage has consistently increased since 2000, reaching 96.1% in 2011. This coverage is close to 100% in the Southeast, South, and Midwest regions. In the North and Northeast regions, some federative units have coverage above 90% and others between 80% and 90%. The percentage of deaths with ill-defined basic causes has been declining over the years, and the South and Midwest regions have the lowest percentage of deaths from ill-defined basic causes (4.5% and 4.4%, respectively)^27^. According to data from the DATASUS^e^, in the period under study, there was a more than 80% decrease in the number of deaths from ill-defined causes in the country, and this decrease was more visible in the Northeast region.

The trends regionally observed may be being influenced by regional inequalities in the recording of mortality data and should be interpreted with caution, e.g. the reduced underreporting of records in regions characterized by poor quality of medical care may be influencing the observed increase in trends. In this sense, the growth trend would rather reflect an improvement in the quality of information than an increase in mortality.

An analogous effect can be observed in the decreasing trends in the rates of cancer of unspecific parts of the mouth and pharynx. Cancer mortality for specific sites that were not correctly diagnosed and recorded may actually be increasing the proportion of deaths from cancer of unspecified sites in the death record set. In the period of 2002 to 2013, 30.6% of the cases of death were classified as neoplasms of unspecified sites of mouth and pharynx corresponding to codes C06 and C14 of the International Classification of Diseases. The decreasing trend in this specific group may also be influenced by improvements in the definition of the specific site in the cause of death.

In addition to the quality of the data obtained in the information systems, the statistical results of a trend analysis are also influenced by the number of years analyzed. In this study, we should proceed with caution in the interpretations of the trends given the relatively short historical series.

An important aspect is the ability to compare the results of the rates obtained with previous studies that use other populations for standardization. However, the new standard used was adopted by the World Health Organization (WHO) to remove the effects of proportional distribution of different populations in the world, from historical events, wars, famine, etc. This translates this new standard into a neutral population for international comparisons and analyses^13^.

Despite the limitations specific to the study, our results show that the trends are either stable or decreasing. Boing et al.^6^ have observed an increase in oropharyngeal and hypopharyngeal cancer mortality, which are anatomical sites that presented stability and decline in our study, respectively. In the aforementioned study, sites with a decreasing trend, such as lip, tongue, floor of mouth, and palate, began to show stability in the period of 2002 to 2013. The stabilization of the observed rate is the result of long-term changes and indicates improvements in the timely diagnosis of oral and pharyngeal cancer and less exposure to risk factors. The different behavior of the trends in each macroregion of the country reflects socioeconomic differences, access to health services, and improvement of information systems that impact on the incidence, mortality, and lethality of this disease.

## CONCLUSION

Pharyngeal cancer mortality presented a decrease, whereas oral cancer mortality presented stability in the period studied. Oral cancer presented an increasing trend in the Northeast region and a decreasing trend in the Southeast region, while pharyngeal cancer decreased in the Southeast, South, and Midwest regions. The cancer mortality rates for other and ill-defined sites in the lip, oral cavity, and pharynx showed a decline between 2002 and 2013. Considering the observed increase in pharyngeal cancer mortality in Brazil, observed in the period of 1979 to 2002, we highlight the decrease observed in the period of 2002 to 2013 for cancer mortality in this anatomical site and the stability presented by the other sites.

## Footnotes

a International Agency for Research on Cancer. Globocan 2012: estimated cancer incidence, mortality and pevalence worlwide. Lyon: IARC; 2012 [cited 2017 Oct 29]. Available from: http://globocan.iarc.fr

b Instituto Nacional de Câncer. Observatório da Política Nacional de Controle do Tabaco. Rio de Janeiro: INCA; s.d. [cited 2017 Oct 29]. Available from: http://www.inca.gov.br/observatoriotabaco/

c Ministério da Saúde (BR), Departamento de Informática do SUS-DATASUS. Sistema de Informações sobre Mortalidade - SIM. Brasília (DF); s.d. [cited 2017 Oct 25]. Available from: http://datasus.saude.gov.br/sistemas-e-aplicativos/eventos-v/sim-sistema-de-informacoes-de-mortalidade

d Instituto Brasileiro de Geografia e Estatística. Censo Demográfico 2010. Rio de Janeiro: IBGE; s.d. [cited 2017 Oct 25]. Available from: https://censo2010.ibge.gov.br/resultados.html

e Ministério da Saúde (BR), Departamento de Informática do SUS - DATASUS. Informações de saúde: estatistícas vitais. Brasília (DF); c.2017 [cited 2017 Sep 30]. Available from: http://datasus.saude.gov.br/informacoes-de-saude/tabnet/estatisticas-vitais
